# One-Carbon Metabolism Biomarkers and Cognitive Decline in the Very Old: The Newcastle 85+ Study

**DOI:** 10.1016/j.jamda.2017.05.008

**Published:** 2017-09-01

**Authors:** Nuno Mendonça, Antoneta Granic, John C. Mathers, Carmen Martin-Ruiz, Keith A. Wesnes, Chris J. Seal, Carol Jagger, Tom R. Hill

**Affiliations:** a School of Agriculture, Food, and Rural Development, Newcastle University, Newcastle-upon-Tyne, UK; b Newcastle University Institute for Ageing, Newcastle-upon-Tyne, UK; c Human Nutrition Research Centre, Newcastle University, Newcastle-upon-Tyne, UK; d Institute of Cellular Medicine, Newcastle University, Newcastle-upon-Tyne, UK; e Institute of Neuroscience, Newcastle University, Newcastle-upon-Tyne, UK; f NIHR Newcastle Biomedical Research Centre in Ageing and Chronic Disease, Newcastle University and Newcastle-upon-Tyne NHS Foundation Trust, Newcastle-upon-Tyne, UK; g Wesnes Cognition Ltd, Streatley-on-Thames, UK; h Department of Psychology, Northumbria University, Newcastle-upon-Tyne, UK; i Medical School, University of Exeter, Exeter, UK; j Institute of Health and Society, Newcastle University, Newcastle-upon-Tyne, UK

**Keywords:** ‘Aged, 80 and over’, cognition, folate, B12, homocysteine

## Abstract

**Objectives:**

Although the biological rationale for the association between folate, vitamin B12, and homocysteine with cognitive function seems plausible, conflicting results have been reported. This study aimed to determine the associations between 1-carbon (1-C) metabolism biomarkers (folate, vitamin B12, and homocysteine), and cognitive impairment at baseline and the rate of cognitive decline over 5 years in the very old.

**Design:**

The Newcastle 85+ Study was a prospective longitudinal study of people 85 years old and followed over 5 years in Northeast England.

**Setting:**

Community-dwelling and institutionalized.

**Participants:**

The analytical sample included 765 very old participants with 1-C metabolism biomarkers and cognitive measures.

**Measurements:**

Global cognition was measured by the Standardized Mini-Mental State Examination (SMMSE) at baseline, and at 3 and 5 years of follow-up and, attention-specific cognition with the Cognitive Drug Research (CDR) System at baseline, and at 1.5 and 3.0 years of follow-up. Baseline red blood cell folate (RBC folate), plasma vitamin B12, and total homocysteine (tHcy) concentrations were determined by immunoassay. Linear mixed models were used to estimate the associations between quartiles of 1-C metabolism biomarkers and cognition over 3 (CDR) and 5 years (SMMSE).

**Results:**

Compared with participants in the lowest quartile of RBC folate concentrations (<612 nmol/L), those in the highest quartile of RBC folate concentrations (>1280 nmol/L) had 1 more point on the SMMSE at baseline (β = +1.02, SE = 0.43, *P* = .02). Those in quartile 4 of tHcy (>21.4 μmol/L) had 1 point less in the SMMSE at baseline than those in the lowest quartile (<13.5 μmol/L) (β = −1.05, SE = 0.46, *P* = .02). Plasma vitamin B12 was not predictive of global or attention-specific cognition at baseline and at follow-up. None of the 1-C metabolism biomarkers except tHcy was associated with the rate of decline in attention scores over 3 years.

**Conclusion:**

RBC folate and tHcy, but not plasma vitamin B12, were associated with better global cognition in the very old at baseline but were not predictive of rate of decline over 5 years.

There are now 46.8 million people with dementia worldwide, and that number is predicted to reach 74.7 million by 2030.^1^ The Cognitive Function and Ageing Study (CFAS) II estimated that there were 670,000 older adults with dementia in 2011 in England alone.^2^ Despite some evidence of reduction in the prevalence and incidence of dementia,^2^, ^3^ it is predicted that cases of dementia will increase primarily for the following reasons: (1) dementia incidence doubles every 6.3 years after 60 years old^1^; and (2) the population is aging worldwide predominantly because of the rise in the numbers of the very old (85 years and older), the fastest growing age segment in the United Kingdom and most Western societies.^4^ Dementia is one of the most important predictors of disability and poses a major societal challenge.^5^ Because cognitive decline and dementia affect quality of life detrimentally and are currently not treatable, research priorities have focused on preventing or delaying the onset of dementia through modifiable risk factors, such as nutrition.^6^

Folate and vitamin B12 are B vitamins that are central to 1-carbon (1-C) metabolism. Inadequate or aberrant 1-C metabolism may be associated with cognitive function through mechanisms such as hyperhomocysteinemia^7^; reduced synthesis of neurotransmitters, phosphatidylcholine, and pyrimidines^8^; altered DNA methylation patterns^9^; and reduced fatty acid synthesis and incorporation of odd chain fatty acids into the myelin sheath.^10^

Although the biological rationale for associations between folate, vitamin B12, and homocysteine with cognitive function seem plausible, conflicting results have been reported. Folate, vitamin B12, and homocysteine have been associated with cognitive decline in some longitudinal studies,^11^, ^12^, ^13^ but not all,^14^, ^15^, ^16^, ^17^ in some randomized controlled trials (RCTs),^18^, ^19^, ^20^ for one vitamin but not the other, or only for certain cognitive domains. Insufficient follow-up time, small sample size, participants’ age at recruitment, and different cognitive tests used are frequently reported reasons for the conflicting results. Furthermore, studies targeting the very old are lacking. We hypothesized that higher red blood cell (RBC) folate and plasma vitamin B12 concentrations would be associated with better cognitive performance and slower rate of cognitive decline, primarily through homocysteine-lowering effects. This study aimed to determine the associations between RBC folate, plasma vitamin B12, and total homocysteine (tHcy) concentrations at baseline, and cognitive impairment and the rate of cognitive decline in global and attention-specific cognition over 5 years in a large population of the very old who participated in the Newcastle 85+ Study.

## Methods

### Participants

Briefly, the Newcastle 85+ Study is a longitudinal study of health trajectories and outcomes in the very old that approached virtually all people turning 85 in 2006 (born in 1921) in North East England. The recruited cohort was broadly sociodemographically representative of the general UK population and, included institutionalized and cognitively impaired very old adults,^21^ 2 commonly excluded groups. Data were collected on multidimensional health aspects and general practice medical records were reviewed at baseline (2006/2007), 18 months (1.5 years), 36 months (3 years), and 60 months later (5 years)^22^ (Figure A1). Further information is reported elsewhere^21^, ^22^, ^23^, ^24^ (for study questionnaires visit http://research.ncl.ac.uk/85plus).

### Biomarkers of 1-C Metabolism

Forty milliliters of blood were drawn from the antecubital vein between 7:00 and 10:30 am after an overnight fast, and 95% of the samples reached the laboratory within 1 hour. Both RBC folate and plasma vitamin B12 were quantified by chemiluminescence (Microparticle Immunoassay on Abbott ARCHITECT analyzer [Abbot Park, IL]) and tHcy by an Abbot IMx immunoassay at baseline.^25^

### Cognitive Assessment

The Standardized Mini-Mental State Examination (SMMSE) was used to assess global cognitive status at baseline and 3- (36 months) and 5-year (60 months) follow-up (Figure A1). The SMMSE is a short, standardized screening test for cognitive impairment in older adults that ranks global cognitive function from 0 to 30. Individuals with SMMSE scores ≥26 were considered as healthy and ≤25 as cognitively impaired.^26^ Three automated tests of attention from the Cognitive Drug Research (CDR) System were used to assess cognition at baseline and again after 1.5 (18 months) and 3.0 years (36 months) (Figure A1). The tests were simple reaction time (SRT), which assesses focused attention and concentration; choice reaction time (CRT), which assessed similar abilities in addition to information processing and decision making; and the digit vigilance task (DVT) that assesses the ability to sustain attention.^27^ Using the measures of speed and accuracy from the tasks, 3 validated composite measures were derived: power of attention (PoA), the sum of 3 speed scores that reflects the ability to focus attention and the intensity of concentration; reaction time variability (RTV), which is a sum of the coefficients of variance of the reaction time scores and reflects variations in attention during the tasks; and continuity of attention (CoA), which combines the accuracy scores from CRT and DVT and reflects the ability to sustain attention.^28^, ^29^ All 3 of these composite scores have been validated previously.^30^, ^31^ Higher scores in the SMMSE and the CoA, and lower scores for the PoA and RTV measures reflect superior performance.

### Other Confounders

Whole-blood DNA was extracted fusing a QiaGEN (Hilden, Germany) Amp Maxi DNA Purification Kit and the gene encoding apolipoprotein E (*APOE*) genotyped for common polymorphisms at rs429358 and rs7412 (to provide information on zygosity at ε2, ε3, and ε4 alleles) by Illumina Omni (San Diego, CA) genotyping arrays.^25^ Multidimensional health questionnaires recorded sex, current housing, years of full-time education, depression (Geriatric Depression Scale), physical activity, supplement use,^32^ alcohol intake (confirmed by 2 × 24-hour multiple pass recalls^33^), and smoking status. Medical records held by the general practitioner were reviewed for diagnosed dementia/Alzheimer disease, diabetes type 1 and 2, hypertension, and history of cardiovascular disease (including angina, myocardial infarction, coronary angioplasty, coronary artery bypass graft, atrial fibrillation, atrial flutter, heart failure, pacemaker use, stroke, transient ischemic attack, and carotid endarterectomy). Weight and height were measured and used to calculate body mass index (BMI). Renal impairment was determined by the chronic kidney disease epidemiology collaboration (CKD-EPI) guidelines using sex, ethnicity, serum creatinine, and age.^34^

### Statistical Analysis

Statistical analyses were conducted using SPSS v22.0 (IBM SPSS Statistics, IBM Corporation, Chicago, IL). Normality was assessed with the Shapiro-Wilk test and confirmed with histograms and Q-Q plots. Linearity assumptions were tested with residuals versus predicted values plots. Multicollinearity of confounders was assessed with variance inflation factor, tolerance, and eigenvalues. Normally distributed continuous values are presented as means and SDs, and non-Gaussian–distributed variables as medians and interquartile ranges (IQRs). Categorical data are presented as percentages (with corresponding sample size). Differences between quartiles of RBC folate, plasma vitamin B12, and tHcy were assessed with χ^2^ test for categorical variables, Mann-Whitney *U* test for continuous nonparametric data, and 1-way analysis of variance for parametric variables.

To account for within-person variability and missing values, the longitudinal effects of RBC folate, plasma vitamin B12, and tHcy on cognitive function were assessed by linear mixed models. Random effects terms included both intercept and slopes of SMMSE and attention scores with the time in the study coded as 0 (baseline), 1 (1.5 or 3.0 years), or 2 (3 or 5 years), respectively. Parameters (β coefficients) were estimated by the maximum likelihood method and the model followed an autoregressive heterogeneous covariance structure. RTV was logarithmically (ln) transformed to correct a positive skew and aid convergence. Other attention scores (PoA and CoA) by 1-C biomarkers were not transformed because the distribution of the residuals of each respective linear mixed model was roughly normal and this did not interfere with convergence. An interaction between the biomarker and time was added to assess the rate of cognitive decline.

Binary logistic regression models were fitted to predict cognitive impairment (SMMSE ≤25) at baseline and incident cognitive impairment after 3 and 5 years. All models were adjusted for sex, *APOE* e4 genotype (rs429358 and rs7412), hypertension, diabetes type 1 and 2, history of cardiovascular diseases, depression, alcohol intake, smoking status, physical activity, years of full-time education, BMI, homocysteine in the folate and vitamin B12 models, and renal impairment in the homocysteine models.

In sensitivity analyses, all models were re-run after excluding participants diagnosed with Alzheimer disease/dementia or living in institutions at baseline, use of folic acid/vitamin B12-containing supplements, with RBC folate concentrations >4000 nmol/L, plasma vitamin B12 concentrations >1000 pmol/L, or tHcy concentrations >40 μmol/L. *P* < .05 was considered statistically significant, unless otherwise stated.

## Results

### Population Characteristics by 1-C Metabolism Biomarkers

The 1-C metabolism biomarkers and SMMSE data were available for 752 to 765 participants at baseline, 414 to 450 at 3 years, and 311 to 318 at 5 years (Figure A1). Attention-specific scores (CDR) were available for 705 to 718 participants at baseline, 528 to 538 at 1.5 years, and 391 to 400 at 3.0 years, depending on the 1-C metabolism assay used (Figure A1). The median concentration for tHcy, RBC folate, and plasma vitamin B12 was 16.7 μmol/L (IQR 13.5–21.4), 863 nmol/L (IQR 451–1287), and 232 pmol/L (IQR 170–324), respectively in the Newcastle 85+ Study.^25^ A concentration >15 μmol/L of tHcy is commonly used to define hyperhomocysteinemia, and concentrations <340 nmol/L and <148 pmol/L to define inadequacy of RBC folate and plasma vitamin B12, respectively. Sixty-three percent of the cohort (n = 482) had tHcy concentrations >15 μmol/L, 4% (n = 26) had RBC folate concentrations <340 nmol/L, and 17% (n = 125) had plasma vitamin B12 concentrations <148 pmol/L.^25^ Table 1, Table 2, Table 3 show the population characteristics at baseline and cognitive scores at follow-up by quartile of RBC folate (quartile 1 [Q1]: <612, Q2: 612–870, Q3: 870–1280, Q4: >1280 nmol/L), plasma vitamin B12 (Q1: >170, Q2: 170–232, Q3: 232–325, Q4: >325 pmol/L), and tHcy (Q1: >13.5, Q2: 13.5–16.7, Q3: 16.7–21.4, Q4: >21.4 μmol/L), respectively. There were fewer *APOE* ε4 carriers in quartile 2 (17%) than in other quartiles (28%–32%) of RBC folate (*P* = .002), and fewer individuals in quartile 1 with history of cardiovascular diseases (45%) than in other quartiles (57%–63%) (*P* < .001). As RBC folate increased, so did plasma vitamin B12 and vice-versa. There was an inverse association between tHcy and the other 1-C metabolism biomarkers. There were more men in tHcy quartile 3 (50%) than in other quartiles (*P* < .001). Furthermore, participants in quartile 4 of tHcy concentrations (>21.3 μmol/L) were less physically active and more likely to be renally impaired compared with those in other quartiles (Table 3).

**Table 1:** Population Characteristics and Cognitive Test Scores at Baseline and Follow-up in the Newcastle 85+ Study by Quartile of RBC Folate Concentration

	Q1, <612 nmol/L	Q2, 612–870 nmol/L	Q3, 870–1280 nmol/L	Q4, >1280 nmol/L	*P*∗
Men, % (n)	41 (77)	38 (72)	40 (75)	38 (72)	.86
BMI, mean (SD), kg/m^2^	24.2 (4.3)	24.7 (4.5)	24.3 (4.1)	24.4 (4.3)	.78†
*APOE* ε4 carriers, % (n)	32 (49)	17 (25)	30 (47)	28 (42)	.02
Plasma vitamin B12, pmol/L	201 (135–280)	216 (159–275)	259 (193–371)	278 (205–391)	<.001
tHcy, μmol/L	19.9 (16.3–24.6)	18.3 (14.9–22.9)	15.6 (13.0–19.6)	13.8 (11.1–17.4)	<.001
Alcohol drinkers, % (n)	70 (92)	73 (94)	80 (96)	66 (89)	.10
Smokers, % (n)	9 (16)	4 (8)	4 (8)	5 (10)	.35
Physical activity, high, % (n)	38 (71)	33 (62)	34 (63)	37 (68)	.75
Education ≥12 y, % (n)	11 (20)	11 (20)	14 (25)	14 (26)	.18
History of cardiovascular disease, % (n)	45 (84)	57 (108)	66 (123)	63 (119)	<.001
Diabetes type 1 or 2, % (n)	9 (16)	16 (31)	13 (25)	17 (32)	.08
Hypertension, % (n)	54 (100)	59 (112)	54 (101)	65 (122)	.12
Renal impairment, % (n)	20 (38)	26 (49)	22 (41)	28 (52)	.36
Depression, severe, % (n)	4 (7)	10 (19)	11 (19)	9 (16)	.25
Global cognition (SMMSE)					
Baseline	27 (24–29)	28 (25–29)	28 (25–29)	28 (25–29)	.17
Impaired, ≤25, % (n)	31 (58)	25 (48)	26 (49)	25 (48)	.52
3 y	27 (23–29)	28 (24–29)	26 (23–29)	27 (25–29)	.19
Impaired, ≤25, % (n)	37 (41)	35 (35)	40 (48)	25 (27)	.10
5 y	28 (23–29)	27 (23–29)	27 (23–28)	28 (25–29)	.17
Impaired, ≤25, % (n)	32 (26)	37 (27)	43 (33)	26 (21)	.16
Focused attention (PoA, ms)					
Baseline	1499 (1341–1691)	1494 (1371–1752)	1484 (1360–1719)	1473 (1341–1667)	.54
1.5 y	1546 (1398–1745)	1538 (1377–1740)	1533 (1399–1731)	1515 (1366–1718)	.84
3.0 y	1594 (1409–1777)	1567 (1378–1775)	1550 (1420–1782)	1490 (1367–1688)	.16
Sustained attention (CoA, ms)					
Baseline	87.8 (80.7–91.4)	87.5 (80.8–91.0)	85.7 (77.0–91.0)	87.7 (82.3–91.3)	.20
1.5 y	86.6 (79.4–90.4)	88.0 (81.9–91.6)	87.0 (78.4–90.5)	86.5 (80.8–90.9)	.24
3.0 y	86.7 (81.1–91.0)	86.8 (79.4–90.7)	86.4 (78.6–90.7)	87.0 (82.2–91.5)	.73
RTV					
Baseline	59.1 (50.6–71.4)	61.2 (54.6–71.7)	61.5 (53.2–73.1)	57.6 (50.3–68.6)	.06
1.5 y	60.0 (53.8–71.0)	60.0 (52.1–68.2)	60.2 (52.2–72.0)	57.6 (52.1–67.0)	.68
3.0 y	59.9 (50.8–70.0)	59.8 (49.4–69.6)	61.4 (51.8–72.3)	59.3 (50.3–66.7)	.60

Q, quartile.

Lower scores in the PoA and RTV, and higher scores in CoA reflect better performance. Continuous variables are presented as medians and IQR unless otherwise stated. History of cardiovascular disease includes cardiac, cerebrovascular, and peripheral vascular diseases.

∗ No quartile difference by χ^2^ for categorical or by Kruskal-Wallis test for nonparametric continuous variables.

† No BMI difference by 1-way analysis of variance.

**Table 2:** Population Characteristics and Cognitive Test Scores at Baseline and Follow-up in the Newcastle 85+ Study by Quartile of Plasma Vitamin B12 Concentration

	Q1, <170 pmol/L	Q2, 170–232 pmol/L	Q3, 232–325 pmol/L	Q4, >325 pmol/L	*P*∗
Men, % (n)	42 (79)	41 (77)	39 (74)	35 (66)	.56
BMI, mean (SD), kg/m^2^	25.1 (4.5)	23.8 (4.1)	25.0 (4.3)	23.7 (4.3)	.001†
*APOE* ε4 carriers, % (n)	26 (40)	24 (38)	23 (35)	32 (50)	.30
RBC folate, nmol/L	683 (479–992)	838 (605–1159)	913 (690–1393)	1058 (745–1608)	<.001
tHcy, μmol/L	19.7 (15.9–25.1)	17.3 (14.5–21.8)	15.9 (13.3–19.8)	13.9 (11.1–18.2)	<.001
Alcohol drinkers, % (n)	73 (103)	72 (91)	80 (91)	65 (86)	.07
Smokers, % (n)	6 (11)	5 (10)	6 (12)	5 (9)	.77
Physical activity, high, % (n)	38 (72)	37 (68)	34 (63)	33 (62)	.55
Education ≥12 y, % (n)	10 (19)	13 (23)	14 (27)	12 (22)	.15
History of cardiovascular disease, % (n)	51 (96)	60 (113)	62 (117)	57 (108)	.10
Diabetes type 1 or 2, % (n)	12 (23)	18 (33)	12 (22)	13 (25)	.32
Hypertension, % (n)	58 (111)	54 (100)	61 (114)	59 (110)	.55
Renal impairment, % (n)	23 (44)	27 (51)	23 (44)	22 (41)	.65
Depression, severe, % (n)	8 (14)	8 (14)	10 (18)	9 (15)	.90
Dementia/Alzheimer, % (n)	6 (12)	8 (14)	8 (15)	6 (11)	.83
Global cognition (SMMSE)					
Baseline	28 (25–29)	27 (25–29)	28 (25–29)	28 (25–29)	.73
Impaired ≤25, % (n)	28 (53)	26 (49)	27 (50)	27 (51)	.99
3 y	27 (23–29)	27 (25–29)	27 (24–29)	27 (24–29)	.94
Impaired ≤25, % (n)	40 (48)	32 (35)	31 (33)	35 (35)	.47
5 y	27 (22–29)	27 (24–29)	27 (23–28)	28 (24–29)	.95
Impaired ≤25, % (n)	38 (33)	35 (28)	32 (24)	33 (23)	.83
Focused attention (PoA, ms)					
Baseline	1502 (1351–1723)	1497 (1370–1710)	1482 (1356–1676)	1467 (1342–1705)	.75
1.5 y	1537 (1370–1848)	1519 (1390–1799)	1548 (1402–1694)	1506 (1373–1725)	.95
3.0 y	1503 (1361–1744)	1560 (1389–1778)	1555 (1425–1732)	1547 (1405–1757)	.69
Sustained attention (CoA, ms)					
Baseline	87.8 (80.7–91.6)	86.8 (81.5–90.9)	87.5 (18.7–91.7)	86.6 (78.5–90.8)	.54
1.5 y	86.8 (81.2–90.9)	87.3 (82.0–90.8)	87.9 (79.6–91.0)	85.8 (78.9–90.7)	.63
3.0 y	86.9 (81.7–91.3)	87.5 (81.0–90.4)	88.3 (81.1–91.8)	85.3 (79.6–90.0)	.09
RTV					
Baseline	60.9 (52.0–70.4)	60.2 (51.0–70.3)	59.6 (52.2–69.6)	58.9 (51.2–72.2)	1.00
1.5 y	60.0 (51.2–67.7)	60.1 (53.7–70.8)	60.4 (52.1–68.3)	57.3 (51.1–68.8)	.45
3.0 y	59.9 (49.5–67.4)	59.8 (51.5–71.6)	59.2 (51.3–67.8)	60.9 (50.6–71.5)	.74

Q, quartile.

Lower scores in PoA and RTV, and higher scores in CoA reflect better performance. Continuous variables are presented as medians and IQR unless otherwise stated. History of cardiovascular disease includes cardiac, cerebrovascular. and peripheral vascular diseases.

∗ No quartile difference by χ^2^ for categorical or by Kruskal-Wallis test for nonparametric continuous variables.

† No BMI difference by 1-way analysis of variance.

**Table 3:** Population Characteristics and Cognitive Test Scores at Baseline and at Follow-up in the Newcastle 85+ Study by Quartile of tHcy Concentration

	Q1, <13.5 μmol/L	Q2, 13.5–16.7 μmol/L	Q3, 16.7–21.4 μmol/L	Q4, >21.4 μmol/L	*P*∗
Men, % (n)	32 (61)	32 (62)	50 (96)	43 (83)	<.001
BMI, mean (SD), kg/m^2^	24.3 (4.4)	24.3 (4.3)	24.9 (4.5)	24.5 (4.3)	.44†
*APOE* ε4 carriers, % (n)	23 (35)	28 (46)	29 (44)	26 (40)	.70
RBC folate, nmol/L	1272 (896–1748)	940 (675–1279)	779 (573–1084)	680 (477–898)	<.001
Plasma vitamin B12, pmol/L	297 (225–430)	230 (185–303)	225 (161–293)	186 (134–262)	<.001
Alcohol drinkers, % (n)	69 (86)	73 (101)	80 (102)	68 (90)	.14
Smokers, % (n)	5 (9)	3 (5)	7 (14)	8 (15)	.01
Physical activity, high, % (n)	38 (72)	34 (65)	38 (72)	31 (58)	.02
Education ≥12 y, % (n)	15 (28)	13 (25)	12 (22)	9 (17)	.57
History of cardiovascular disease, % (n)	56 (106)	58 (112)	59 (113)	59 (113)	.88
Diabetes type 1 or 2, % (n)	14 (27)	13 (25)	13 (25)	16 (31)	.80
Hypertension, % (n)	56 (107)	52 (99)	59 (113)	63 (121)	.14
Renal impairment, % (n)	8 (15)	15 (29)	22 (41)	51 (98)	<.001
Depression, severe, % (n)	9 (16)	11 (19)	5 (9)	10 (18)	.52
Dementia/Alzheimer, % (n)	9 (17)	8 (15)	6 (12)	7 (13)	.77
Global cognition (SMMSE)					
Baseline	28 (26–29)	28 (26–29)	28 (25–29)	27 (24–29)	.01
Impaired, ≤25, % (n)	24 (46)	23 (45)	29 (55)	32 (61)	.19
3 y	27 (24–29)	27 (24–29)	27 (25–29)	27 (22–29)	.46
Impaired, ≤25, % (n)	31 (35)	30 (34)	35.0 (43)	43 (42)	.17
5 y	28 (25–28)	27 (23–29)	28 (25–29)	27 (23–29)	.80
Impaired, ≤25, % (n)	32 (28)	38 (31)	28 (24)	41 (26)	.32
Focused attention (PoA, ms)					
Baseline	1450 (1326–1662)	1486 (1354–1723)	1476 (1351–1659)	1517 (1409–1753)	.03
1.5 y	1532 (1371–1731)	1519 (1371–1711)	1513 (1379–1724)	1601 (1444–1782)	.13
3.0 y	1492 (1369–1689)	1546 (1383–1749)	1564 (1442–1786)	1559 (1406–1800)	.25
Sustained attention (CoA, ms)					
Baseline	87.8 (80.7–91.7)	87.6 (81.4–91.3)	87.3 (79.2–90.8)	86.3 (78.3–91.3)	.44
1.5 y	86.0 (81.7–90.8)	88.2 (82.7–91.5)	88.0 (81.5–90.8)	85.1 (76.8–90.2)	.02
3.0 y	86.8 (80.2–91.8)	87.5 (81.0–91.7)	87.0 (81.3–90.7)	86.3 (76.5–90.0)	.35
RTV					
Baseline	57.1 (50.8–67.3)	61.8 (52.0–72.1)	60.3 (51.5–72.8)	61.2 (53.0–71.8)	.08
1.5 y	60.5 (53.1–71.1)	57.0 (50.8–67.0)	59.4 (52.9–67.8)	62.9 (54.0–71.4)	.04
3.0 y	55.8 (49.6–67.8)	60.8 (52.0–68.6)	60.7 (51.2–72.5)	61.8 (51.6–69.7)	.34

Q, quartile.

Lower scores in PoA and RTV, and higher scores in CoA reflect better performance. Continuous variables are presented as medians and IQR unless otherwise stated. History of cardiovascular disease includes cardiac, cerebrovascular, and peripheral vascular diseases.

∗ No quartile difference by χ^2^ for categorical or by Kruskal-Wallis test for nonparametric continuous variables.

† No BMI difference by 1-way analysis of variance.

### Longitudinal Associations With Cognitive Performance

Participants lost to follow-up (died or unable to complete any cognitive test; Figure A1) after 5 years (n = 501) had slightly higher median tHcy (*P* = .01) and plasma vitamin B12 concentrations (*P* = .05); were more likely to be *APOE* ε4 carriers (*P* = .01); were less likely to drink alcohol (*P* < .001); less physically active (*P* < .001); more likely to have a history of CVD (*P* = .003), diabetes type 1 and 2 (*P* = .01), and Alzheimer disease/dementia (*P* < .001); and more likely to live in institutions (*P* < .001) at baseline compared with those who had SMMSE data 5 years later. Furthermore, more participants lost to follow-up during every phase were more cognitively impaired than those who continued on the study.

Table 4 shows the associations between RBC folate, plasma vitamin B12, and tHcy quartiles and attention-specific and global cognitive decline over 3 and 5 years, respectively, using linear mixed models.

**Table 4:** Association Between Folate, Vitamin B12, and Homocysteine and Attention-Specific and Global Cognitive Decline in the Newcastle 85+ Study

Biomarker	Change Over Time, β (SE); *P*	Intercept, β (SE); *P*	Biomarker × Time, β (SE); *P*
Global cognitive function (SMMSE)			
RBC folate, nmol/L			
Q2 (612–870)	−1.69 (0.18); <.001	+0.57 (0.42); .17	−0.38 (0.51); .46
Q3 (870–1280)		+0.61 (0.42); .15	−0.88 (0.49); .08
Q4 (>1280)		+1.02 (0.43); .02	+0.13 (0.51); .80
Plasma vitamin B12, pmol/L			
Q2 (170–232)	−1.68 (0.18); <.001	+0.62 (0.42); .14	+0.50 (0.49); .30
Q3 (232–325)		−0.15 (0.43); .73	−0.55 (0.50); .27
Q4 (>325)		+0.54 (0.43); .21	+0.15 (0.51); .77
tHcy, μmol/L			
Q2 (13.5–16.7)	−1.68 (0.18); <.001	−0.53 (0.41); .20	+0.26 (0.49); .60
Q3 (16.7–21.4)		−0.74 (0.43); .08	+0.96 (0.50); .06
Q4 (>21.4)		−1.05 (0.46); .02	+0.35 (0.53); .50
Focused attention (PoA, ms)			
RBC folate, nmol/L			
Q2 (612–870)	+104 (15); <.001	+19 (62); .76	−10 (43); .82
Q3 (870–1280)		+20 (63); .75	+98 (42); .02
Q4 (>1280)		−45 (63); .48	+27 (43); .52
Plasma vitamin B12, pmol/L			
Q2 (170–232)	+105 (15); <.001	−95 (62); .13	+40 (42); .34
Q3 (232–325)		−41 (62); .52	+26 (43); .55
Q4 (>325)		−114 (64); .07	+35 (43); .42
tHcy, μmol/L			
Q2 (13.5–16.7)	+105 (15); <.001	+108 (61); .08	−110 (42); .01
Q3 (16.7–21.4)		+53 (63); .40	−93 (42); .03
Q4 (>21.4)		+81 (68); .23	−100 (44); .02
Sustained attention (CoA, ms)			
RBC folate, nmol/L			
Q2 (612–870)	−1.26 (0.30); <.001	0.91 (1.20); .45	−0.34 (0.84); .69
Q3 (870–1280)		−1.27 (1.22); .30	−0.45 (0.83); .58
Q4 (>1280)		+1.00 (1.23); .41	−1.07 (0.84); .21
Plasma vitamin B12, pmol/L			
Q2 (170–232)	−1.27 (0.30); <.001	+0.76 (1.21); .53	−0.45 (0.81); .58
Q3 (232–325)		−1.00 (1.22); .41	−0.62 (0.83); .45
Q4 (>325)		−0.32 (1.24); .80	−0.75 (0.84); .37
tHcy, μmol/L			
Q2 (13.5–16.7)	−1.27 (0.30); <.001	+0.38 (1.18); .75	+1.65 (0.82); .04
Q3 (16.7–21.4)		+1.58 (1.22); .20	+2.19 (0.83); .01
Q4 (>21.4)		+0.04 (1.32); .98	+1.58 (0.87); .07
RTV			
RBC folate, nmol/L			
Q2 (612–870)	+0.021 (0.008); .01	+0.017 (0.026); .52	−0.037 (0.022); .09
Q3 (870–1280)		+0.043 (0.027); .10	+0.001 (0.021); .97
Q4 (>1280)		−0.019 (0.027); .49	+0.028 (0.022); .20
Plasma vitamin B12, pmol/L			
Q2 (170–232)	+0.021 (0.008); .01	+0.015 (0.026); .58	+0.001 (0.021); .95
Q3 (232–325)		+0.034 (0.027); .20	−0.017 (0.021); .44
Q4 (>325)		+0.010 (0.027); .72	−0.015 (0.022); .49
tHcy, μmol/L			
Q2 (13.5–16.7)	+0.021 (0.008); .01	+0.016 (0.026); .53	+0.035 (0.028); .22
Q3 (16.7–21.4)		+0.018 (0.027); .49	+0.030 (0.030); .31
Q4 (>21.4)		+0.034 (0.029); .24	+0.042 (0.031); .18

Q, quartile.

For all models, Q1 (<612 nmol/L RBC folate, <170 pmol/L plasma vitamin B12, and <13.5 tHcy, respectively) was used as the reference (0.00). Models are adjusted for alcohol intake, smoking status, *APOE* genotype (rs429358 and rs7412), sex, education, BMI, depression, hypertension, diabetes type 1 and 2, history of cardiovascular diseases, and physical activity. RBC folate and plasma vitamin B12 models were additionally adjusted for tHcy and the homocysteine model for renal impairment. Higher scores in the SMMSE and CoA, and lower scores in PoA and RTV tests represent better performance.

Participants in the highest quartile of RBC folate concentrations had 1 more point on the SMMSE at baseline than those in quartile 1 (β = +1.02, SE = 0.43, *P* = .02) after adjustment for sex, alcohol intake, smoking status, *APOE* genotype, education, BMI, depression, diabetes type 1 and 2, hypertension, history of cardiovascular disease, physical activity, and tHcy (Table 4). Plasma vitamin B12 concentration measured at baseline was not predictive of global cognition (SMMSE) in the nonadjusted (data not shown) or fully adjusted models. Conversely, participants in the highest quartile of tHcy had 1 point less on the SMMSE score than those in the lowest quartile at baseline (β = −1.05, SE = 0.46, *P* = .02). Folate, vitamin B12, and tHcy were not predictive of any attention-specific measures (PoA, CoA, and RTV) over 3 years (Table 4).

### Rate of Cognitive Decline by 1-C Metabolism Biomarkers

All domains of cognitive performance declined significantly (lower scores of SMMSE and CoA and higher scores in PoA and RTV) with time. SMMSE and CoA decreased on average by 1.68 (SE = 0.18, *P* < .001) and by 1.27 ms (SE = 0.3, *P* < .001) respectively, PoA and RTV (ln) increased by 105 ms (SE = 15, *P* < .001) and by 0.021 (SE = 0.008, *P* = .01) respectively, for every phase. There were no significant changes in the rate of global cognitive decline (SMMSE) by quartiles of RBC folate, plasma vitamin B12, and tHcy (Table 4). There were also no significant differences in the rate of decline in attention-specific scores (CDR) except for a slower decline in focused attention (PoA) (eg, Q4 vs Q1: β = −100, SE = 44, *P* = .02) and a trend for slower decline of sustained attention (CoA) (eg, Q4 vs Q1: β = +1.58, SE = 0.87, *P* = .07) in the higher quartiles of tHcy concentration (Table 4 and Figures A2–A4).

### Prevalent and Incident Cognitive Impairment

Binary logistic regression models, adjusted for the same covariates, showed that participants in Q4 of tHcy had an increased risk (odds ratio [OR] 2.15, confidence interval [CI] 1.12–4.11, *P* = .02) of prevalent cognitive impairment (defined as SMMSE ≤25 points in SMMSE) than those in Q4, but not for incident impairment after 3 years (data not shown) or after 5 years (Table A1).

### Sensitivity Analysis

Sensitivity analyses excluding those with diagnosed Alzheimer disease/dementia (n = 52–56), living in institutions at baseline (n = 61–62), use of folic acid or vitamin B12 containing supplements (n = 35–37), with RBC folate concentrations >4000 nmol/L (n = 6), plasma vitamin B12 concentrations >1000 pmol/L (n = 16), or tHcy concentrations >40 μmol/L (n = 16) did not generally change the results. However, higher homocysteine quartiles were significantly associated with higher scores in PoA (poorer performance) over 5 years when participants diagnosed with dementia or Alzheimer disease at baseline were excluded. The interaction between quartiles of tHcy concentration and time for focused attention (PoA) was no longer present when these same participants were excluded from the analysis.

## Discussion

### Main Findings

This study showed that in the very old, higher quartiles of RBC folate and lower quartiles of tHcy concentration measured at baseline were associated with better global cognition as measured by the SMMSE. In contrast, plasma vitamin B12 was not associated with cognition (global and attention) at any time points. RBC folate, plasma vitamin B12, and tHcy concentration were not predictive of the rate of decline in SMMSE and attention, except for higher tHcy concentration, which was associated with slower decline in focused attention (PoA).

### Other Studies

Results of folate and global cognition and/or specific cognitive domains in prospective cohort studies are frequently in disagreement. This study confirmed findings from studies that showed that folate, as measured by serum folate or RBC folate, was associated with global cognition or specific cognitive domains,^11^, ^12^, ^13^, ^14^, ^35^, ^36^, ^37^ whereas others did not find these relationships.^16^, ^17^, ^38^ However, apart from Leiden 85-Plus, these studies focused on younger populations. Voluntary folic acid fortification of cereal grains products introduced in 1996 in the United States and changed into mandatory fortification in 1998 might partly explain the lack of associations between folate and cognitive performance in some studies, as it is difficult to find overt folate deficiency.^16^ The median (IQR) RBC folate concentration in our cohort was 863 (IQR 451–1287) nmol/L and only 4% (n = 26) had concentrations <340 nmol/L. With reference to cutoffs derived from younger populations, this RBC folate concentration might be considered replete, which also could explain the lack of associations with cognitive decline. The choice of biomarkers to assess folate status also deserves attention, as some studies that did not find an association^16^, ^38^ used serum folate and not RBC folate, which more closely reflects long-term status.

In contrast with our findings, some studies have reported that holotranscobalamin or plasma vitamin B12 concentration was predictive of cognitive decline,^13^, ^38^, ^39^, ^40^, ^41^ whereas others failed to find an association.^12^, ^14^, ^16^, ^17^, ^35^, ^36^, ^39^ As with our study, some studies,^11^, ^12^, ^13^, ^35^, ^38^ but not all,^14^, ^17^, ^36^, ^40^ have reported that tHcy concentration was associated with global cognition or specific cognitive domains measured over time. The Leiden 85-Plus study had a very similar design to the Newcastle 85+ study and included 599 adults aged 85 years at baseline. Participants were followed for 4 years and global cognitive function was assessed by the Mini-Mental State Examination and attention was measured by the Stroop test. Mooijaart et al^14^ reported findings similar to our study. Low serum folate and high tHcy, but not serum vitamin B12, were associated with cognitive impairment cross-sectionally, but these biomarkers did not predict cognitive decline or rate of cognitive decline over 4 years in the Leiden 85-Plus. In the same study, tHcy concentration was associated with attention at baseline but not folate or vitamin B12 and not longitudinally.^14^

The lack of specificity of the assay used for plasma vitamin B12 may explain the lack of associations between cognitive decline and vitamin B12 in the Newcastle 85+ Study and the Leiden 85-Plus. The assay assesses not only the vitamin's active form, holotranscobalamin, which makes up 20% to 30% of plasma vitamin B12, but also the other 70% to 80% bound to haptocorrin and considered inert.^42^ The use of more recent and more robust markers of vitamin status, such as holotranscobalamin and methylmalonic acid (MMA), might have yielded different results. In fact, a review of longitudinal cohort studies of vitamin B12 status and cognitive decline found that, in all studies in which holotranscobalamin and MMA had been used, associations between vitamin B12 status and cognitive decline, dementia, or Alzheimer disease were present.^43^ Those with obvious dementia usually show no improvement when treated with vitamin B12.^44^ In the Newcastle 85+ Study, 9% of the participants had been diagnosed with dementia/Alzheimer disease at baseline, which might have skewed the results and partly explained the lack of association. However, in sensitivity analyses, when we excluded participants with dementia from the models, the lack of association between plasma vitamin B12 and cognitive performance at baseline and at follow-up remained. Very high plasma vitamin B12 concentrations have been found in patients with inflammatory, liver, and kidney diseases, which could underestimate the association with cognitive performance.^45^ Therefore, models were re-run excluding those with plasma vitamin B12 concentrations greater than 1000 pmol/L, but the results were similar. Perhaps the most convincing data so far from RCTs on the association between B vitamins and cognition comes from the VITACOG studies.^20^ This RCT included 168 adults older than 70 with mild cognitive impairment who were either assigned to a treatment arm (daily dose of 0.8 mg folic acid, 0.5 mg vitamin B12, and 20 mg vitamin B6) or a placebo and followed for 2 years. Smith et al^20^ found that brain atrophy was 53% slower in those treated with B vitamins than with placebo, but only in those with tHcy concentrations >13 μmol/L at baseline.

In the present study, the rate of decline in focused attention (PoA) and sustained attention (CoA) was slower in higher quartiles of tHcy. This is counterintuitive, as lower concentrations of tHcy are associated with better health outcomes, including slower rate of cognitive decline.^20^ However, this is likely an effect of terminal decline, as homocysteine is a strong predictor of mortality in the very old,^46^, ^47^ which may have selected more cognitively robust survivors in higher quartiles of tHcy. The rate of decline in focused (PoA) and sustained attention (CoA) was no longer associated with tHcy if individuals diagnosed at baseline with dementia or Alzheimer disease were excluded.

### Strengths and Limitations

In the Newcastle 85+ Study, SMMSE and attention-specific CDR System tests were applied at 3 different time points but only over the period of 5 and 3 years, respectively. Such follow-up periods may be too short to detect relationships with 1-C metabolism biomarkers.^43^ However, due to the advanced age of the participants, it is likely that cognitive decline was far more rapid than in younger populations, and the SMMSE as well as the CDR System would be able to detect this decline over 5 and 3 years, respectively. Although there is no gold standard on how to evaluate cognitive performance, the SMMSE has a well-known ceiling effect of 30 points and a cognitive decline from above 30 points would not be captured.^48^ Also for this reason, it has been suggested that the SMMSE might not be sensitive enough to detect subtle cognitive changes typical of most nutritional interventions.^49^ Cognitive function is a strong predictor of mortality. Therefore, because participants with cognitive impairment were more likely to be lost to follow-up due to death, this could potentially dilute the cognitive decline results.

In the Newcastle 85+ Study, 28% of the participants were cognitively impaired (SMMSE ≤25) at baseline. Reverse causality cannot be fully excluded for any cross-sectional associations among homocysteine, folate, and cognitive performance. Finally, RBC folate, plasma vitamin B12, and tHcy measurements were available at baseline only, which may not reflect their status at other time points and lead to underestimation of their association with cognitive function.^38^

## Conclusion

The results suggest that tHcy and RBC folate, but not plasma vitamin B12, concentrations are predictive of better global cognition in the very old at baseline but not rate of cognitive decline. These results warrant confirmation by RCTs with enough follow-up time and sufficient power to detect cognitive decline.

## Figures and Tables

**Table A1:** Association Between Folate, Vitamin B12, and Homocysteine and Prevalent Cognitive Impairment at Baseline and Incident Cognitive Impairment After 5 Years (SMMSE≤25) in the Newcastle 85+ Study

	Prevalent Cognitive Impairment				Incident Cognitive Impairment			
	Model 1		Model 2		Model 1		Model 2	
	OR (95% CI)	*P*	OR (95% CI)	*P*	OR (95% CI)	*P*	OR (95% CI)	*P*
RBC folate, nmol/L								
Q2 (612–870)	0.77 (0.44–1.33)	.35	0.73 (0.40–1.31)	.29	1.68 (0.68–4.14)	.26	1.68 (0.61–4.65)	.31
Q3 (870–1280)	0.74 (0.43–1.28)	.29	0.71 (0.38–1.30)	.26	2.40 (1.02–5.65)	.05	2.59 (0.96–7.02)	.06
Q4 (>1280)	0.84 (0.49–1.45)	.53	0.73 (0.40–1.34)	.31	1.10 (0.42–2.87)	.84	0.84 (0.27–2.67)	.88
Plasma vitamin B12, pmol/L								
Q2 (170–232)	0.80 (0.46–1.40)	.44	0.64 (0.35–1.18)	.16	1.15 (0.52–2.53)	.73	1.41 (0.56–3.53)	.46
Q3 (232–325)	1.06 (0.62–1.81)	.84	1.03 (0.57–1.87)	.93	0.64 (0.26–1.54)	.32	0.61 (0.23–1.66)	.34
Q4 (>325)	0.89 (0.51–1.54)	.66	0.67 (0.36–1.23)	.19	0.85 (0.36–2.03)	.72	1.12 (0.40–4.11)	.84
Total homocysteine, μmol/L								
Q2 (13.5–16.7)	0.99 (0.56–1.75)	.96	1.05 (0.57–1.96)	.87	1.12 (0.50–2.54)	.79	1.71 (0.68–4.30)	.26
Q3 (16.7–21.4)	1.18 (0.67–2.09)	.57	1.40 (0.75–2.60)	.29	0.79 (0.34–1.88)	.60	0.97 (0.37–2.56)	.96
Q4 (>21.4)	1.55 (0.89–2.69)	.13	2.15 (1.12–4.11)	.02	1.10 (0.46–2.64)	.83	1.81 (0.63–5.25)	.27

Quartile 1 (<612 nmol/L RBC folate, <170 pmol/L plasma vitamin B12, and <13.5 tHcy, respectively) was used as the reference category (1.00) for all models. Model 1 is unadjusted, Model 2 is adjusted for alcohol intake, smoking status, *APOE* genotype (rs429358 and rs7412), sex, education, BMI, depression, hypertension, diabetes type 1 and 2, history of cardiovascular diseases, and physical activity. RBC folate and plasma vitamin B12 models were additionally adjusted for tHcy and the homocysteine model for renal impairment.

## Supplementary Data

Fig. A1 Flowchart of the Newcastle 85+ Study. GP, general practitioner; vit., vitamin.

Fig. A2 Linear decline in (A) global cognition (SMMSE), (B) focused attention (PoA), (C) sustained attention (CoA), and (D) RTV by quartiles of RBC folate concentration. β estimates were derived from linear mixed models adjusted for alcohol intake, smoking status, *APOE* genotype (rs429358 and rs7412), sex, education, BMI, depression, hypertension, diabetes type 1 and 2, history of cardiovascular diseases, physical activity, and tHcy. Quartile 1 was used as the reference (0.00). Higher scores in the SMMSE and CoA and, lower scores in PoA and RTV tests represent better performance. Ln, natural logarithm; ms, milliseconds.

Fig. A3 Linear decline in (A) global cognition (SMMSE), (B) focused attention (PoA), (C) sustained attention (CoA), and (D) RTV by quartiles of plasma vitamin B12 concentration. β estimates were derived from linear mixed models adjusted for alcohol intake, smoking status, *APOE* genotype (rs429358 and rs7412), sex, education, BMI, depression, hypertension, diabetes type 1 and 2, history of cardiovascular diseases, physical activity, and tHcy. Quartile 1 was used as the reference (0.00). Higher scores in the SMMSE and CoA, and lower scores in PoA and RTV tests represent better performance. Ln, natural logarithm.

Fig. A4 Linear decline in (A) global cognition (SMMSE), (B) focused attention (PoA), (C) sustained attention (CoA), and (D) RTV by quartiles of tHcy concentration. β estimates were derived from linear mixed models adjusted for alcohol intake, smoking status, *APOE* genotype (rs429358 and rs7412), sex, education, BMI, depression, hypertension, diabetes type 1 and 2, history of cardiovascular diseases, physical activity, and renal impairment. Quartile 1 was used as the reference (0.00). Higher scores in the SMMSE and CoA and, lower scores in PoA and RTV tests represent better performance. Ln, natural logarithm.

## Footnotes

Supplementary data related to this article can be found online at http://dx.doi.org/10.1016/j.jamda.2017.05.008.
