# Supplementary figures and images for: One-Carbon Metabolism Biomarkers and Cognitive Decline in the Very Old: The Newcastle 85+ Study
Source: J Am Med Dir Assoc. 2017 Sep 1;18(9):806.e19–27. doi: 10.1016/j.jamda.2017.05.008 (PMC5576913; doi:10.1016/j.jamda.2017.05.008)

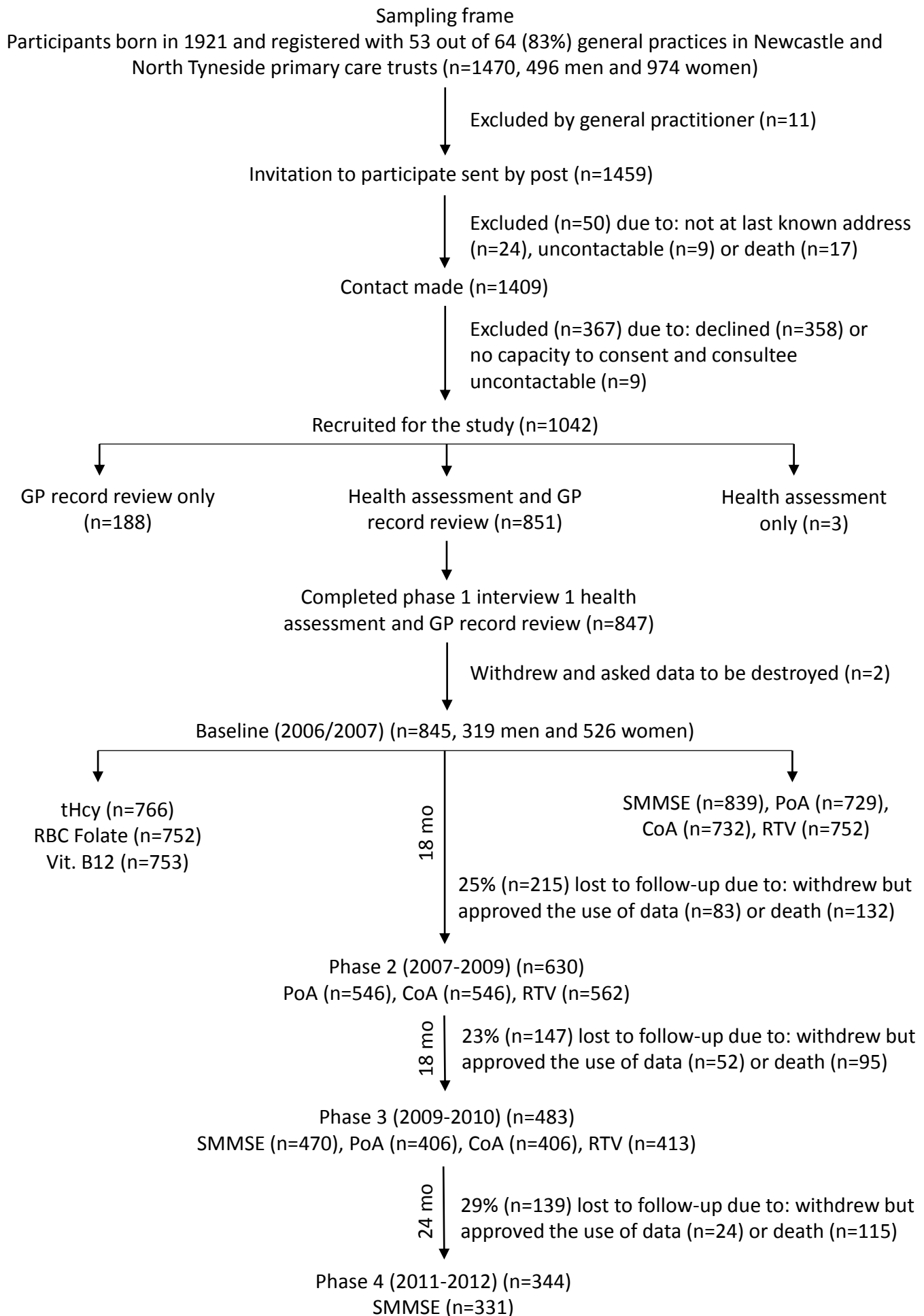

Supplement: Fig. A1 — Flowchart of the Newcastle 85+ Study. GP, general practitioner; vit., vitamin. [file mmc1.pdf]

**A.**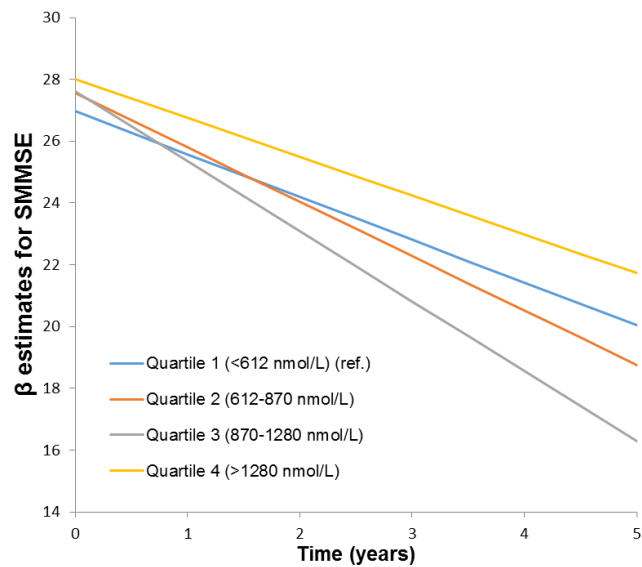**B.**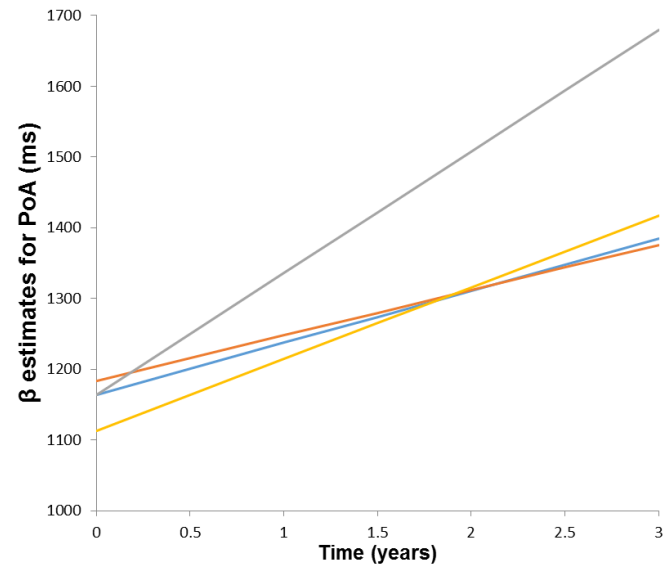**C.**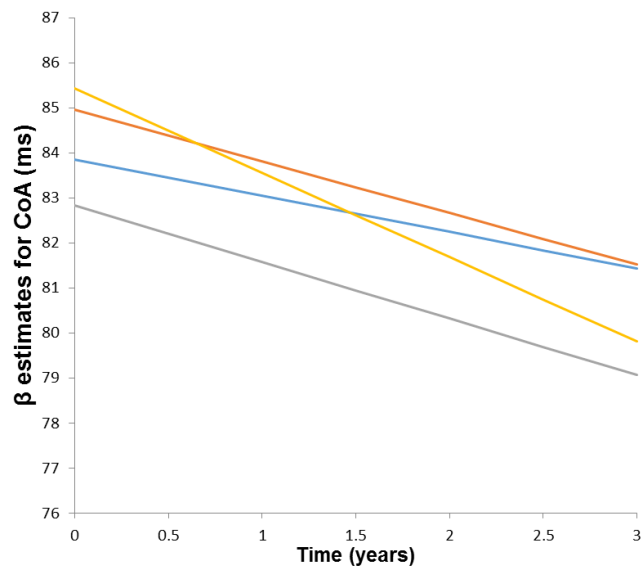**D.**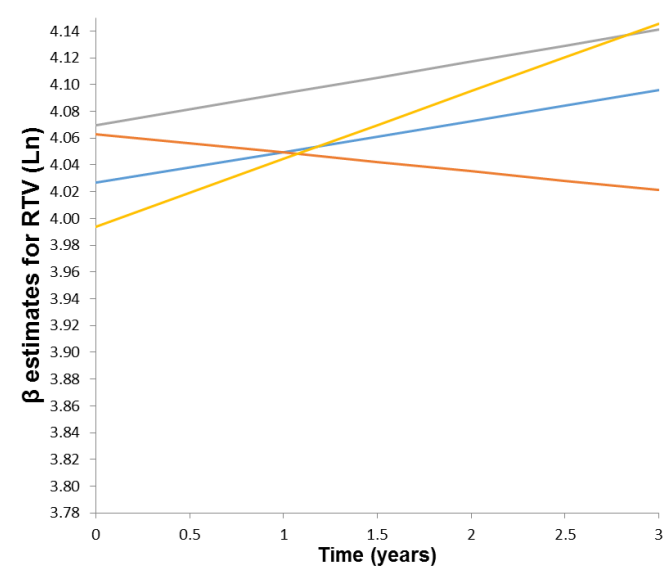

Supplement: Fig. A2 — Linear decline in (A) global cognition (SMMSE), (B) focused attention (PoA), (C) sustained attention (CoA), and (D) RTV by quartiles of RBC folate concentration. β estimates were derived from linear mixed models adjusted for alcohol intake, smoking status, APOE genotype (rs429358 and rs7412), sex, education, BMI, depression, hypertension, diabetes type 1 and 2, history of cardiovascular diseases, physical activity, and tHcy. Quartile 1 was used as the reference (0.00). Higher scores in the SMMSE and CoA and, lower scores in PoA and RTV tests represent better performance. Ln, natural logarithm; ms, milliseconds. [file mmc2.pdf]

**A.**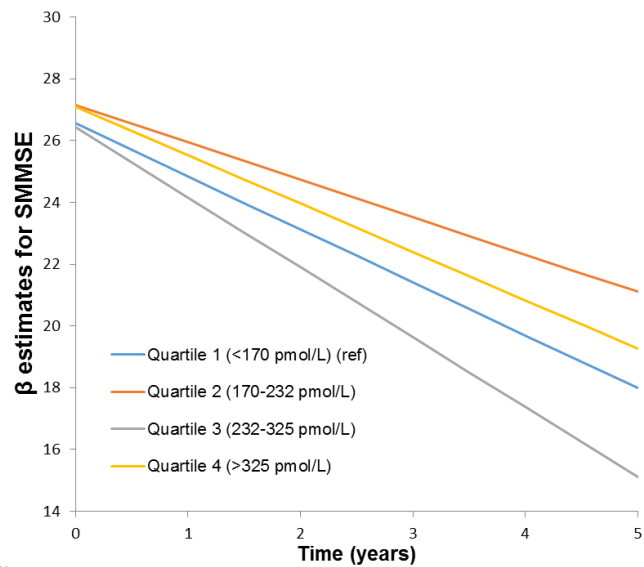**B.**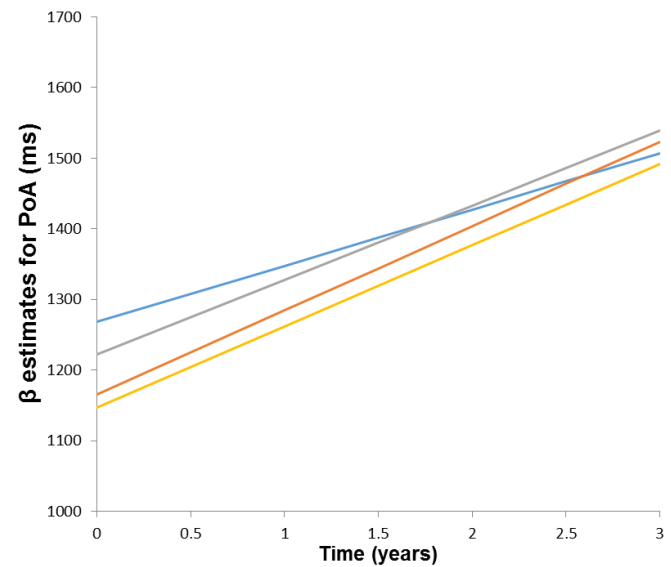**C.**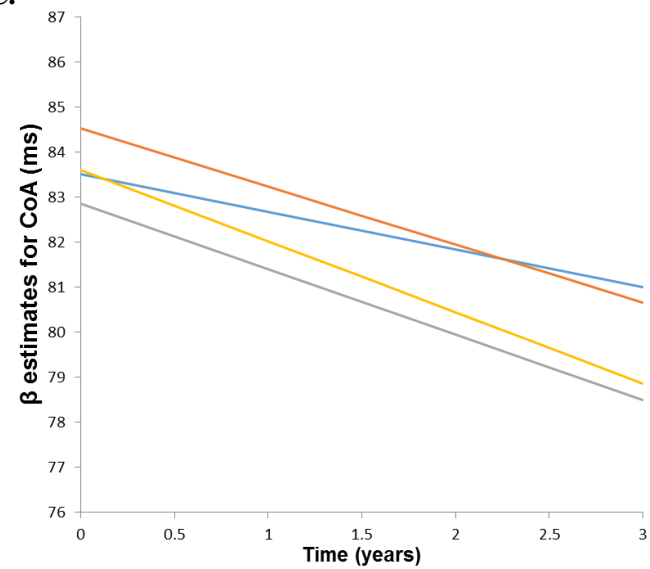**D.**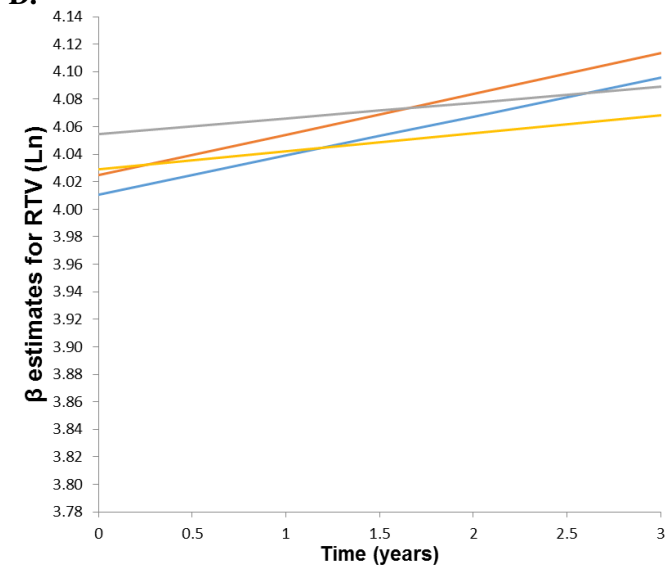

Supplement: Fig. A3 — Linear decline in (A) global cognition (SMMSE), (B) focused attention (PoA), (C) sustained attention (CoA), and (D) RTV by quartiles of plasma vitamin B12 concentration. β estimates were derived from linear mixed models adjusted for alcohol intake, smoking status, APOE genotype (rs429358 and rs7412), sex, education, BMI, depression, hypertension, diabetes type 1 and 2, history of cardiovascular diseases, physical activity, and tHcy. Quartile 1 was used as the reference (0.00). Higher scores in the SMMSE and CoA, and lower scores in PoA and RTV tests represent better performance. Ln, natural logarithm. [file mmc3.pdf]

**A.**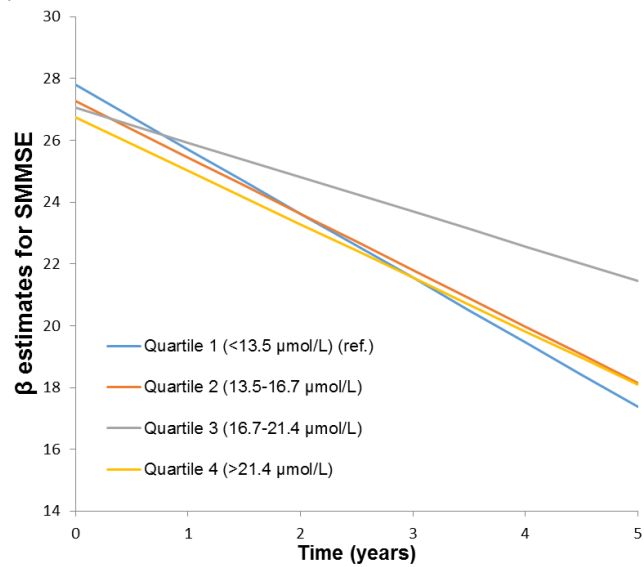**B.**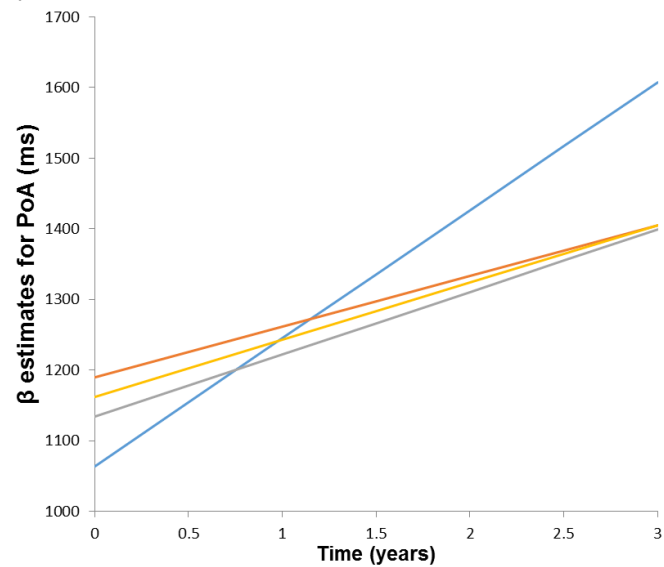**C.**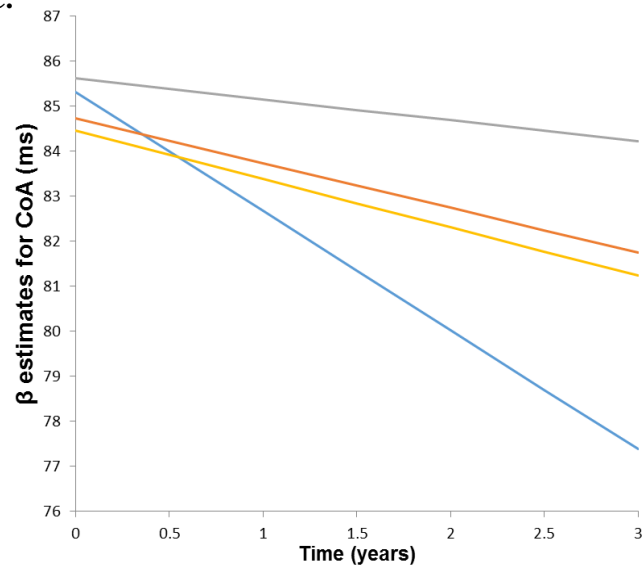**D.**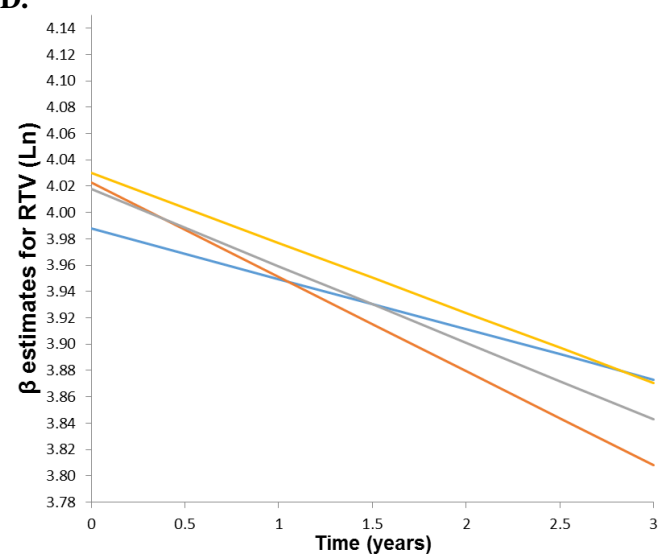

Supplement: Fig. A4 — Linear decline in (A) global cognition (SMMSE), (B) focused attention (PoA), (C) sustained attention (CoA), and (D) RTV by quartiles of tHcy concentration. β estimates were derived from linear mixed models adjusted for alcohol intake, smoking status, APOE genotype (rs429358 and rs7412), sex, education, BMI, depression, hypertension, diabetes type 1 and 2, history of cardiovascular diseases, physical activity, and renal impairment. Quartile 1 was used as the reference (0.00). Higher scores in the SMMSE and CoA and, lower scores in PoA and RTV tests represent better performance. Ln, natural logarithm. [file mmc4.pdf]
